# Frequency and pattern of Paediatric Heart Diseases: Five years experience at The Children’s Hospital, Multan

**DOI:** 10.12669/pjms.36.6.2312

**Published:** 2020

**Authors:** Muhammad Sohail Arshad, Hafiz Muhammad Anwar-ul-Haq, Mudasser Adnan, Arif Zulqarnain

**Affiliations:** 1 Dr. Muhammad Sohail Arshad, FCPS (Paeds Cardiology). Department of Paediatric Cardiology, The Children’s Hospital & the Institute of Child Health, Multan, Pakistan; 2 Dr. Hafiz Muhammad Anwar-ul-Haq, FCPS (Paeds Medicine). Department of Paediatric Cardiology, The Children’s Hospital & the Institute of Child Health, Multan, Pakistan; 3 Dr. Mudasser Adnan, FCPS (Paeds Medicine). Department of Paediatric Cardiology, The Children’s Hospital & the Institute of Child Health, Multan, Pakistan; 4 Dr. Arif Zulqarnain, FCPS (Paeds Medicine). Department of Paediatric Cardiology, The Children’s Hospital & the Institute of Child Health, Multan, Pakistan

**Keywords:** Congenital heart disease, Acquired heart disease, Echocardiography, Rheumatic heart disease

## Abstract

**Background & Objectives:**

Heart diseases in paediatric population are considered to be significant contributors to mortality and morbidity. Congenital heart disease (CHD) as well as acquired heart disease (AHD) are frequent causes of hospital admission among children. This study was aimed at finding out frequency and pattern of heart diseases in admitted patients at The Children’s Hospital, Multan.

**Methods:**

This study is a retrospective chart review of five years at Paediatric Cardiology Department of The Children’s Hospital and The Institute of Child Health, Multan, Pakistan, from January 2015 to December 2019. Children aged one month to 15 years, admitted as a diagnosed case of heart disease on the basis of echocardiography were included.

**Results:**

Out of a total of 4115 confirmed cases of heart disease admitted during the study period, 3250 (79.0%) were CHD while 865 (21.0%) were AHD. Overall, 2861 (69.5%) patients were aged less than one year. VSD followed by ASD were the commonest acyanotic heart lesion seen among 927 (28.5%) and 644 (19.8%) cases while TOF was the commonest cyanotic type heart lesion found in 396 (12.2%). Rheumatic heart disease (RHD) was the commonest type of AHD, seen in 330 (38.2%) cases followed by acute myocarditis found in 230 (26.6%) cases.

**Conclusion:**

Burden of heart diseases is rising in our region. VSD, ASD and TOF were the most common types of CHDs while RHD and acute myocarditis were the most frequent types of AHDs.

## INTRODUCTION

Heart diseases in paediatric population are considered to be significant contributors to mortality and morbidity. Congenital Heart Disease (CHD) is a frequent form of heart diseases found among children and adults. CHD also forms a major proportion of all major congenital malformations affecting 2 to 3% of neonates while prevalence of CHD varies from 3-10/1000 live births all around the globe.^1^,^2^ In Pakistan about 40,000 children are born with a congenital heart defect annually.^3^ Incidence was found to be 8.2/1000 live births in a study in China,^4^ and 8.1/1000 live births in a study in Atlanta.^5^ In developing countries the burden of congenital heart defects is increasing day by day because of increase in risk factors and etiological factors for these defects.^6^ Mortality among CHD cases is high while majority of the cases report late to cardiac facilities when complications have already taken place which further adds to already high mortality rates.^7^,^8^

In developed countries early detection and proper treatment has increased the survival rate and has decreased mortality from 80% to 20% causing an increase in the number of adults with CHD.^9^,^10^ Recent local data indicates ventricular septal defect (VSD) to be the most frequent type of acyanotic heart lesion found in 27% of CHD cases while tetrology of Fallot (TOF) has been noted as the most frequent type of cyanotic heart defect seen in 10.9% cases.^11^

Acquired heart disease (AHD) is a condition that affects the heart and its related blood vessels commonly developing during childhood and comprise of illnesses like rheumatic heart disease, myocarditis, cardiomyopathy, bacterial endocarditis and pericarditis.^12^ AHDs are also a frequent cause of hospital admission among children.^13^

In Pakistan, few studies have been published from tertiary care centers looking into frequency and pattern of heart diseases. No data from South Punjab Region of Pakistan is available so this retrospective study analyzing data of last five years about our experience was aimed at finding out frequency and pattern of heart diseases in admitted patients at The Children’s Hospital, Multan. The results of this study will give us insight about the burden of heart diseases in our region while it will further help us devising appropriate strategies to reduce morbidity and mortality associated with it.

## METHODS

This study is a retrospective chart review done at Paediatric Cardiology Department of The Children’s Hospital and The Institute of Child Health, Multan, Pakistan, from January 2015 to December 2019. Approval from Institutional Ethical Committee was taken for this study (IRB/201-2019).

Children aged one month to 15 years, admitted as a diagnosed case of heart disease on the basis of echocardiography were included. Patients visiting outpatient department without being admitted, or critically ill patients (at risk of death) who could not be sent for echocardiography, were excluded.

Presence of CHD or AHD was confirmed with the help of echocardiography done by consultant Paediatric Cardiologist. We were focused on analyzing total number and types of CHD and AHD cases along with gender and age distribution. All the study data was recorded on a predesigned proforma. Data was handled and analyzed using SPSS version 21.0. Data was represented in terms of frequency and percentage.

## RESULTS

Out of a total of 4115 confirmed cases of heart disease admitted during the study period, 3250 (79.0%) were CHD while 865 (21.0%) were AHD. Amongst CHD cases, 1812 (55.8%) were male and 1438 (44.2%) female. Among AHD cases, 467 (54.0%) were male and 398 (46.0%) female.

Most of the children having CHD, 2709 (83.4%) were having age less than one year, 387 (11.9%) between one to five years, 107 (3.3%) between 5 to 10 years while remaining 47 (1.4%) were above 10 years of age. Among cases having AHD, 152 (17.6%) were below one year of age, 215 (24.9%) from one to five years, 218 (25.2%) between 5 to 10 years while remaining 280 (32.4%) were above 10 years of age.

VSD followed by ASD were the commonest acyanotic heart lesion seen among 927 (28.5%) and 644 (19.8%) cases while TOF was the commonest cyanotic type heart lesion found in 396 (12.2%). Rheumatic heart disease (RHD) was the commonest type of AHD, seen in 330 (38.2%) cases followed by acute myocarditis found in 230 (26.6%) cases.

**Fig.1:**
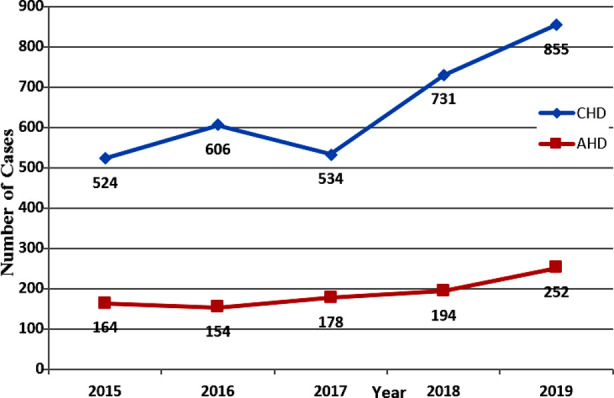
Pattern of Heart Diseases from Year 2015 to 2019.

## DISCUSSION

In the developed countries, advancement in health sciences is helping in relatively early diagnosis and awareness among cases suffering with heart diseases but in countries like Pakistan, timely diagnosis and management of heart diseases among paediatric population is still a challenge. Not much work has been done locally to uncover the epidemiological and clinical aspects of children suffering with heart disease. CHD are known to be the commonest congenital defects found with high rates of mortality during 1^st^ year of life.^11^ It has been observed that patterns and prevalence of CHD vary around the world. This study was aimed to give insight about the burden of heart diseases in our region.

In the present study, we noted that majority of the children with heart diseases were male. A local study conducted by Mohammad N et al^13^ also noted that 55.3% of the cases having heart diseases were male. Similar pattern showing male predominance has been observed by other local researchers as well^11^ while a study from India showed that 66% of the study participants with CHD were male.^14^

The age for the detection of heart disease vary greatly around the world because of so many factors involved.^15^ Many cases of heart diseases are commonly missed especially minor defects which are often asymptomatic. We noted that most of the children (83.4%) having CHD were below one year of age. Karthiga S et al from India^14^ noted 69.7% of the children with CHD to be below one year of age. Shah GS et al.^16^ in their findings from a tertiary care hospital noted 55.5% of the cases with CHD were noted to have age below one years. Khan I and Colleagues^17^ in a local study analyzing echocardiographically confirmed cases of CHD found 71% of their cases to be less than 1 year of age which is quite similar to the current findings.

In the current work, VSD followed by ASD were the commonest acyanotic heart lesions seen among 927 (28.5%) and 644 (19.8%) cases while TOF was the commonest cyanotic type heart lesion found in 396 (12.2%). Our results are quite consistent with those found by a study conducted at Agha Khan University Hospital Karachi,^18^ where they noted VSD to be the most frequent acyanotic defect while TOF was found to be the most frequent cyanotic defect. In another local study from Hazara^19^ noted 61.4% of the study participants with CHD to be having VSD while TOF and ASD (8.8% each) were the other most common defects. A retrospective analysis of 3072 cases from Peshawar^20^ also noted VSD to be the commonest form of CHD. Another local study^21^ noted TOF as most frequent type of CHD (24.4%) which is quite different to what so many others noted. Kapoor R and Coworkers^22^ from India noted VSD to be the most common type of CHD, seen in 21.3% of their children while ASD was noted among 18.9%. Globally, VSD is found to be the commonest acyanotic CHD seen among 25 to 30% of all CHD cases.^23^

**Table-I:** Frequency of Various Types of CHD and Distribution with regards to Gender and Age (n=3250).

CHD Types, n (%)	Gender		Age			

	Male (%)	Female (%)	<1	1-5	5-10	>10
Tetrology of Fallot, 396 (12.2%)	214 (54.0%)	182 (46.0%)	331 (83.6%)	48 (12.1%)	12 (3.0%)	5 (1.3%)
TGA with VSD with PS, 131 (4.0%)	75 (57.3%)	56 (42.7%)	121 (9.2%)	6 (4.6%)	4 (3.2%)	0
TGA with VSD with PH, 106 (3.3%)	62 (58.4%)	44 (41.6%)	96 (90.6%)	8 (7.5%)	1 (0.9%)	1 (0.9%)
VSD, 927 (28.5%)	511 (55.1%)	416 (44.9%)	859 (92.7%)	52 (5.6%)	11 (1.2%)	5 (0.5%)
ASD, 644 (19.8%)	382 (59.3%)	262 (40.7%)	562 (87.3%)	64 (9.9%)	12 (1.9%)	6 (0.9%)
Complete AVSD, 134 (4.1%)	71 (53.0%)	63 (43.0%)	102 (76.1%)	20 (14.9%)	8 (6.0%)	4 (3.0%)
PDA, 356 (11.0%)	184 (51.7%)	172 (48.3%)	238 (66.9%)	79 (22.2%)	30 (8.4%)	9 (2.5%)
Pulmonary atresia, 62 (1.9%)	36 (58.0%)	26 (42.0%)	44 (71.0%)	10 (16.10%)	5 (8.0%)	3 (4.8%)
Tricuspid atresia, 71 (2.2%)	43 (60.6%)	28 (39.4%)	49 (69.0%)	12 (16.9%)	6 (8.5%)	4 (5.6%)
Truncus arteriosis Type I, 28 (0.9%)	16 (57.1%)	12 (42.9%)	21 (75.0%)	4 (14.3%)	3 (10.7%)	0 (0%)
Cardiac TAPVC, 37 (1.1%)	20 (54.1%)	17 (45.9%)	26 (70.1%)	11 (29.7%)	0 (0%)	0 (0%)
Supra cardiac TAPVC, 37 (1.1%)	19 (51.3%)	18 (48.7%)	27 (73.0%)	10 (27.0%)	0 (0%)	0 (0%)
Ebstein Anomally, 25 (0.8%)	14 (56.0%)	11 (44.0%)	19 (76.0%)	4 (16.0%)	1 (4.0%)	1 (4.0%)
Complex Heart Disease, 84 (2.6%)	44 (52.4%)	40 (47.6%)	56 (66.7%)	18 (21.4%)	6 (7.1%)	4 (4.8%)
Others, 212 (6.5%)	121 (57.1%)	91 (42.9%)	158 (74.5%)	41 (19.3%)	8 (3.8%)	5 (2.4%)
Total, 3250	1812 (55.8%)	1438 (44.2%)	2709 (83.4%)	387 (11.9%)	107 (3.3%)	47 (1.4%)

**Table-II:** Frequency of various types of AHD and distribution with Regards to gender and age (n=865).

Diagnosis, n (%)	Gender		Age (years)			

	Male (%)	Female (%)	<1	1to5	5to10	>10
Rheumatic Heart Disease, 330 (38.2%)	178 (53.9%)	152 (46.1%)	0 (0%)	0 (0%)	132 (40.0%)	198 (60.0%)
Acute myocarditis, 230 (26.6%)	124 (53.9%)	106 (46.1%)	70 (30.4%)	146 (63.4%)	9 (3.9%)	5 (2.2%)
Dilated Cardiomyopathy, 90 (10.4%)	51 (56.7%)	39 (43.3%)	45 (50.0%)	22 (24.4%)	14 (15.6%)	9 (10.0%)
Infective Endocarditis, 29 (3.4%)	16 (55.2%)	13 (44.8%)	0 (0%)	7 (24.1%)	18 (62.1%)	4 (13.8%)
Secondary Pulmonary Hypertension, 84 (9.7%)	44 (52.4%)	40 (47.6%)	0 (0%)	19 (22.6%)	29 (34.5%)	36 (42.9%)
Hypertrophic Cardiomyopathy, 8 (0.9%)	4 (50.0%)	4 (50.0%)	4 (50.0%)	3 (37.5%)	1 (12.5%)	0
Restrictive Cardiomyopathy, 6 (0.7%)	4 (66.7%)	2 (33.3%)	0 (0%)	2 (33.3%)	3 (50.0%)	1 (16.7%)
Tuberculous pericardial Effusion, 15 (1.7%)	8 (53.3%)	7 (46.7%)	0 (0%)	2 (13.3%)	6 (40.0%)	7 (46.7%)
Anemic Heart Failure, 73 (8.4%)	38 (52.1%)	35 (47.9%)	33 (45.2%)	14 (19.2%)	6 (8.2%)	20 (27.4%)
Total, 865	467 (54.0%)	398 (46.0%)	152 (17.6%)	215 (24.9%)	218 (25.2%)	280 (32.4%)

In the present study, rheumatic heart disease was the commonest type of AHD, seen in 330 (38.2%) cases followed by acute myocarditis 230 (26.6%). Dilated cardiomyopathy was observed in 90 (10.4%). Cardiomyopathies could be congenital as well as acquired.^13^ We considered cardiomyopathies to be AHD in our study. Our findings stand similar to another local study from Lahore^24^ where it was noted that among AHDs, RHD was the most common 44.5% followed by myocardial diseases in 27.7%. Our findings were also in accordance with regional data.^25^ High presence of RHD could be because of poverty, unhygienic living environment, high illiteracy rate and large family size in our country.^24^

Variation in pattern and frequency of CHD may be attributed to variation in detection methods, difference in standards of healthcare facilities as well as difference in genetics and other environmental factors.

### Limitations of this study

Being a retrospective study, we were limited to a set protocol regarding data inclusion and analysis which might affect interpretation of the results. Majority of our study participant are comprised of young infants so there is a need to establish a long-term study to see early functional limitations in these individuals. We were unable to collect any follow up data so follow up data about preschool and school going age will further shed light about various issues faced in these years in these children. Future studies should be planned to further analyze outcomes with regards to different lesion types.

## CONCLUSION

Burden of heart diseases is rising in our region. VSD, ASD and TOF were the most common types of CHDs while RHD and acute myocarditis were the most frequent types of AHDs. Diagnosis of heart diseases in the early age is pointing towards improvement in healthcare facilities. Population based studies on a large scale are needed to estimate the burden and pattern of heart diseases in Pakistan. Majority of the patients with CHD need surgical correction or intervention in cath lab. As the hospital admission percentage of such patients less than 1 year of age is quite high, the need of the day is to establish more and more cardiac surgery centers capable of doing surgeries in infancy.

### Authors’s Contribution:

**MSA:** Conceived, methodology, proof reading and responsible for data’s authenticity and integrity.

**HMA:** Drafting, data analysis.

**MA:** Literature Review, Introduction.

**AZ:** Data Interpretation, Discussion.
